# CAR-T cell therapy in ovarian cancer: from the bench to the bedside

**DOI:** 10.18632/oncotarget.19929

**Published:** 2017-08-04

**Authors:** Xinxin Zhu, Han Cai, Ling Zhao, Li Ning, Jinghe Lang

**Affiliations:** ^1^ Department of Obstetrics and Gynecology, Peking Union Medical College Hospital, Peking Union Medical College, Chinese Academy of Medical Sciences, Beijing, China; ^2^ Department of Obstetrics and Gynecology, Institute for Wound Research, University of Florida, Gainesville, Florida, USA

**Keywords:** chimeric antigen receptor, T lymphocyte, immunotherapy, ovarian neoplasms, toxicity

## Abstract

Ovarian cancer (OC) is the most lethal gynecological malignancy and is responsible for most gynecological cancer deaths. Apart from conventional surgery, chemotherapy, and radiotherapy, chimeric antigen receptor-modified T (CAR-T) cells as a representative of adoptive cellular immunotherapy have received considerable attention in the research field of cancer treatment. CARs combine antigen specificity and T-cell-activating properties in a single fusion molecule. Several preclinical experiments and clinical trials have confirmed that adoptive cell immunotherapy using typical CAR-engineered T cells for OC is a promising treatment approach with striking clinical efficacy; moreover, the emerging CAR-Ts targeting various antigens also exert great potential. However, such therapies have side effects and toxicities, such as cytokine-associated and “on-target, off-tumor” toxicities. In this review, we systematically detail and highlight the present knowledge of CAR-Ts including the constructions, vectors, clinical applications, development challenges, and solutions of CAR-T-cell therapy for OC. We hope to provide new insight into OC treatment for the future.

## INTRODUCTION

Ovarian cancer (OC) is the second most common gynecological malignancy in the United States, with 21,290 new cases in 2015, and the most frequent cause of gynecological cancer-related mortality, with 14,180 estimated deaths in the same year [1]. Because of the insidious nature, limited screening tools, and nonspecific symptoms of OC, most patients are not diagnosed with OC until it has reached an advanced International Federation of Gynecology and Obstetrics stage [2]. The standard treatment for OC involves cytoreductive surgery, when necessary, followed by a combination of platinum- and taxane-based chemotherapy [3]. Although an initial response to chemotherapy occurs in more than 80% of patients, the cancer recurs in most patients, with an 18-month median time to progression [4, 5]. Thus, novel and effective therapeutic approaches are urgently required to achieve a long-term favorable clinical prognosis for patients with OC. Adoptive T-cell immunotherapy is one of the most robust immunotherapy methods for treating cancers [6], and the early T-cell transfer trials enrolling patients with OC have yielded promising results [7, 8]. Chimeric antigen receptor–modified T (CAR-T)-cell therapy is a representative variant of adoptive T-cell immunotherapy and has received considerable attention in the research and treatment of cancers. CAR-T-cell immunotherapy involves using gene transfer technology to reprogram patients’ T cells to express CARs, thereby directing the cytotoxic potential of T lymphocytes against cancer cells [9]. Numerous notable studies have revealed that CAR-T-cell immunotherapy is an effective therapeutic strategy for cancers including OC [7, 8, 10, 11]. However, this therapy has side effects and related toxicities. In this systematic review, we present an overview of the biological understanding, clinical applications, and challenges of CAR-T-cell therapy in OC.

## STRUCTURE OF CARS

**Figure 1:**
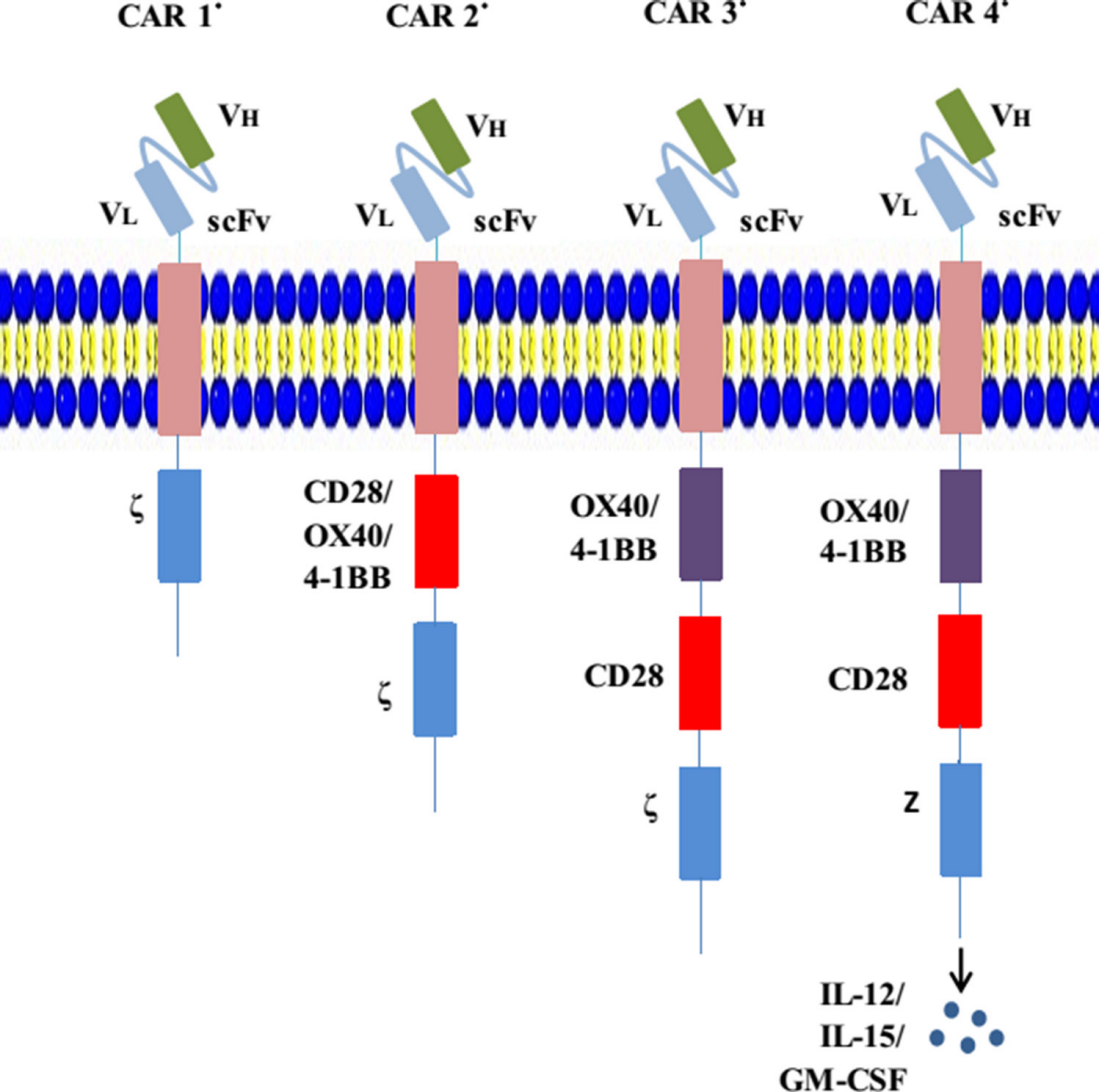
Evolution of CARs CAR 1° represents the first-generation CARs; an scFv links the CD3ζ or FcεRIγ in the transmembrane region. CAR 2° represents the second-generation CARs; costimulatory molecules such as CD28 are engineered to the signal-transduction region. CAR 3° represents the third-generation CARs; they contain two costimulatory domains. CAR 4° (also called TRUCK) represents the fourth-generation CARs; they are additionally modified using a constitutive or inducible expression cassette for a transgenic protein (e.g., a cytokine), which is released by the CAR-T-cell to modulate T-cell response.

A chimeric antigen receptor (CAR) is a type of genetically engineered receptor. The structure of CARs comprises four parts: an extracellular antigen recognition region with single-chain variable fragments (scFvs), which derive from an antigen-specific mAb and recognize and bind specific tumor-related antigens independent of major histocompatibility complex (MHC) molecule restriction; an extracellular stalk (hinge) domain that typically comprises either Fc domains or the spacer domain from a cluster of differentiation 4 (CD4) and CD8 [12, 13]; a transmembrane domain that is usually derived from CD8, CD3-ζ, CD4, OX40, and H2-K^b^ [14]; and an intracellular signaling tail including a signal-transduction component of a T-cell receptor (TCR) (e.g., CD3ζ immunoreceptor tyrosine-based activation motif domain) and/or a costimulatory receptor (e.g., CD28, CD27, 4-1BB, or OX40) (Figure 1) [15]. The scFvs, formed by a combination of antibody heavy- and light-chain amino acid sequences with a short peptide linker, are attached to the hinge region, where they act as extracellular antigen-binding domains [16]. The variable region binds antigens and is capable of enormous combinatorial diversity, enabling the recognition of a myriad of specific molecular conformations [17]. The useful feature of an scFv comes from its “single chain” nature, which enables its incorporation into a CAR vector construct and efficient transduction into T cells. An scFv is characterized by its immunogenicity, affinity, specificity, and binding epitope [18]. With scFvs, CARs can specifically engage targets and trigger downstream signals.

CAR structures have evolved and there are now four generations used in clinical practice, the main distinctions between which are the presence of diverse costimulatory molecules (Figure 1). The primal CAR-T cell comprises a scFv fraction and signal-transduction domain (CD3ζ or FcεRIγ), which endows the modified T cell with activation and homing abilities. Although preclinical experiments and early phase clinical trials using first-generation CAR-T cells have demonstrated that they engaged their target antigens and are generally safe, the infused CAR-T cells exhibited short persistence and minimal efficacy [19, 20]. To improve the persistence of CAR-T-cell cytotoxicity, second-generation CAR-T cells have been designed on the basis of primal generation with the addition of one intracellular costimulatory domain, including the CD28 molecule or members of the tumor necrosis factor receptor family such as CD27, 4-1BB (CD137), and OX-40, which was discovered to increase the resultant persistence and cytotoxicity [21–23]. Receptors containing both CD3ζ and CD28 (or CD27, 4-1BB, or OX-40) are the prototypes for second-generation CAR-T cells, which are currently being rapidly expanded to various arrays of receptors with diverse functional properties. More recently, third-generation CARs (triple-fusion receptors) comprising CD3ζ and two costimulatory molecules have been developed, which further enhance cytotoxicity durability compared with dual-fusion receptors [24, 25]. Newly generated fourth-generation CARs, also named TRUCK T cells, were engineered to produce cytokines, particularly interleukin (IL)-12, which can regulate the antitumor microenvironment. IL-15 and granulocyte-macrophage colony-stimulating factor (GM-CSF) also contribute to this strategy [16]. A more recent study, which generated allogeneic universal T cells deficient of both programmed death 1 (PD-1) and cytotoxic T-lymphocyte antigen (CTLA-4) pathways (inhibitory pathways for immune escape), was attempted using human lymphocytes *in vitro* [26]. Moreover, a double-antigen CAR-T was also proposed and applied in an animal model to reduce CAR-T-related toxicities [27].

## CAR CONSTRUCT TRANSDUCTION: VIRAL AND NONVIRAL APPROACHES

Various genetic methods are used to transfer a specific gene into mouse or human T lymphocytes. These methods, including viral and nonviral methods, differ in the expression levels and stability of the modified CAR-T cells. This paper describes frequently used viral approaches—gamma retroviral, lentiviral, adenovirus, and adeno-associated viral vectors—as well as nonviral approaches such as liposomal-mediated gene transfer, messenger RNA–mediated gene transduction and Sleeping Beauty transposon/transposase system. (Table 1).

**Table 1:** Frequently used approaches to transduction in tumors

	Viral vectors				Nonviral vectors		
Features	RV	LV	AV	AAV	liposomal	mRNA	transposon/ transposase
Structure	ssRNA	ssRNA	dsDNA	ssDNA			
Infected cell	dividing cells	dividing and quiescent cells	dividing and quiescent cells	dividing and quiescent cells		dividing and quiescent cells	
Integration	Yes	Yes	No	Yes	No	No	poor
Clinical applications	most widely used now				most widely used nonviral vectors		less been applied but have great potential
General advantage	higher infection rate				safety, ability to transfer large size gene, less toxicity		
General chanllege	immunogenicity, carcinogenicity, poor target cell specificity, inability to transfer large size genes				low transfection efficiency, poor transgene expression		
Cost of production	costly and laborious				cheap and relatively simple		

AAV, adeno-associated virus vector; AV, adenovirus vector; dsDNA, double-stranded deoxyribonucleic acid; LV, lentiviral vector; mRNA, messenger ribonucleic acid; RV, retroviral vector; ssDNA, single-stranded deoxyribonucleic acid; ssRNA, single-stranded ribonucleic acid.

### Viral approaches

#### Retroviral vectors (RVs)

In general, viral vectors are more efficient at delivering target genes to cells than physical methods such as direct DNA injection and gene gun technology [28]. The ability of RVs to successfully deliver foreign genes was first reported in 1981 [29]. In OC, the gene therapy approach initially employed was the use of recombinant RVs [30, 31]. RVs are lipid-enveloped particles containing two identical copies of a linear single-stranded RNA genome of length approximately 7–11 kb [32]. The viral protein genes (gag, pol, and env) are removed from retroviruses during the development of the gene delivery carrier. Substitutability and integration are the two principal features of retroviruses. Substitutability refers to the ability for a majority of the retroviral genome to be replaced with a transgene of interest. Integration refers to the permanent integration of the retroviral transgene into the host's genome during cell division [33]. RVs are promising stable and efficient gene transfer systems and are generally employed in OC therapy [34, 35]. However, large-scale use of RVs in clinical practice still involves challenges such as insertional mutagenesis and high titer vector production, which may cause cellular immortalization and neoplastic transformation [36]. Moreover, most of the retroviruses infect only actively dividing cells during cell mitosis [37]. Although this feature may protect normal cells, tumor tissues also contain nondividing cells in the G0 phase. Such cells may escape from the therapy. Therefore, improving vector designs, selecting appropriate cancer types, and elucidating tumor cell biology are crucial issues that must be addressed before the extensive application of RVs in clinics.

### Lentiviral vectors (LVs)

LVs, although sharing many features with RVs derived from oncogenic retroviruses, can also transduce some resting cells *in vivo*; this is because they can pass through intact host nuclear membranes and do not need cell division for integration [28, 38, 39]. This enables LV transduction in a wide range of cells including both dividing and nondividing cells. LVs integrate into the target cell genome stably, leading to the lasting expression of the gene of interest. In an OC model, LVs greatly outperformed RVs in growth-arrested cell transduction, and the transfection efficiency of LVs in OC cells was 10 times higher than that of RVs [39]. This was an expected and important finding because that study was the first to confirm that LVs can infect nonproliferating cells among growth-arrested OC cells. The genomic integration sites of LVs are not related to promoter regions, which can decrease the risk of meaningful insertional mutagenesis [33, 40]. All the aforementioned features render LV systems safer and more efficient for CAR expression and application in cancer immunotherapy. Several applications that use modified CAR-T cells with LV systems are being developed to treat OC [41, 42] and achieve active results.

### Adenoviruses

Adenoviruses are double-stranded DNA viruses that infect both dividing and nondividing cells and can cause a wide range of benign respiratory infections in humans [43, 44]. Defective-competent adenoviral vectors (AVs) were first established by replacing the viral E1 gene with a therapeutic gene. Subsequently, more valid gene carriers were produced by altering more genes in the viral genome, such as the E2 gene [45]. AVs have been extensively used in cancer immunotherapy and successfully developed for selective tumor gene therapy in OC [46–48]. One study reported transfection using AVs to be transient because the adenoviral DNA genome does not integrate into the host genetic material permanently [49]. Therefore, repetitive administration of AVs is required to obtain the desired therapeutic outcome. To overcome the low infection rate of AVs, researchers have explored a class of infective-enhanced AVs—consisting of coxsackie-adenovirus receptor-independent targeting motifs RGD (Ad5.RGD), polylysine (Ad5.pK7), or both (Ad5.RGD.pK7)—for their use in OC gene therapy. These AVs were found to infect OC cell lines with substantially enhanced infectivity. Among the developed AVs, Ad5.RGD.pK7 exhibited the highest efficacy in a subcutaneous tumor model [50].

### Adeno-associated viral vectors (AAVs)

An AAV is a nonpathogenic, single-stranded DNA parvovirus with an inverted terminal repeat (ITR) on the end of each single-stranded DNA genome. An ITR is the only *cis*-acting element required for genome replication and packaging [51]. As a dependovirus, an AAV carries two viral genes, *rep* and *cap*, which are removed in gene therapy. An AAV can infect both dividing cells and quiescent cells [52]. Emerging recombinant AAV (rAAV) gene delivery vectors are produced by deleting the two viral genes *rep* and *cap* and inserting a transgene expression cassette between the two ITRs. Therefore, rAAVs have minimal associated toxicity, which makes them potential tools for delivering a vast range of appropriate transgenes in numerous disease models. Kringle 5 (K5) of human plasminogen is one of the most potent angiogenesis inhibitors. A study investigating the antitumor effects of rAAV-mediated delivery of human-OC-cell K5 gene (a angiogenesis inhibitor) in mouse models reported that a single injection of AAV-K5 inhibited both subcutaneous and intraperitoneal growth of human OC cells [53]. A similar study indicated that an antiangiogenic gene in combination with an rAAV can be used to treat OC growth and dissemination [54]. When evaluating the successful therapeutic outcomes of a gene delivery vector, long-term gene expression and infection efficiency should not be neglected. In addition, when investigating the tremendous potential of AAVs for efficient gene delivery, limiting factors such as internalization, endosomal trafficking, and nuclear import should be considered.

### Nonviral approaches

To address the limitations of viral vectors, such as their safety and the capacity of their transgenic materials, researchers have been encouraged to focus on investigating nonviral vectors as an alternative. In contrast to viral vectors, nonviral systems are easy to produce and have a much lower risk of inflammatory complications [55].

### Liposome-mediated gene transfer

Lipid-based vectors are the most extensively used nonviral gene carriers. In 1980, a study first demonstrated that liposomes composed of the phospholipid phosphatidylserine entrapped and delivered SV40 DNA to monkey kidney cells [56]. Yu et al. [57] revealed that liposome-mediated E1A gene transfer substantially suppressed the growth and dissemination of OC cells that overexpressed HER2/neu in mice. Most of (approximately 70%) these mice survived for more than 365 days, whereas all the mice in the control group, which did not receive the liposome-mediated gene therapy, died within 160 days. This result reveals that liposome-mediated E1A transduction may be a valid immunotherapy approach for human OCs that overexpress HER-2/neu. Cationic lipids are currently widely used for liposomal gene transfer because of their extraordinary potential to condense DNA [58, 59]. In ovarian adenocarcinoma, the cationic liposome DDC [a combination of dioleoyltrimethylaminopropane (DOTAP), 1,2-dioleoyl-3-phosphatidylethanolamine, and cholesterol] is a promising nonviral vector because of its selective high gene transfer ability [60]. Various liposomal formulations have been employed, including DOTAP [61], dioctadecylamidoglycylspermine, and dipalmitoyl phosphatidylethanolamidospermine [62]. Cationic liposomes have been explored *in vitro*, *in vivo*, and also in clinical patients [63]; however, the main difficulty that remains to be resolved is the low transfection efficiency for clinical applications.

### Messenger RNA (mRNA)-mediated gene transduction

*In vitro*–transcribed mRNA-mediated gene delivery has gained special popularity as an alternative to DNA-based nonviral and viral gene transduction methods. An mRNA CAR delivery system does not incur the risk of insertional mutation or potential malignant transformation/genotoxicity of transduced cells compared with viral transduction, which can integrate into the host genome [64]. Moreover, mRNA gene transduction can be used to transduce both quiescent and proliferating cells. A study revealed that transduced CAR-T cells often cause on-target, off-tumor effects in normal tissue because of their ability to achieve stable and prolonged expression [65]. An mRNA delivery system is characterized by transient CAR expression; thus, on-target, off-tumor side effects are self-limited as the RNA CAR degrades. Data have revealed that in an mRNA induction system, the autologous T cells are enriched, expanded, and active for 8–12 days [66, 67]. In several cancer types such as mesothelioma and acute myeloid leukemia, mRNA-transduced CAR-T cells have been reported to generate the expected antitumor effects at both primary and metastatic sites [66, 68].

### *Sleeping Beauty (SB)* transposon/transposase system

The SB transposon/transposase system is another nonviral approach and has been employed in clinical practice to stably insert a CAR to redirect T-cell specificity [69]. The transposon/transposase system has two components: a plasmid carrying the gene of interest (transposon) and another plasmid encoding the transposase [70]. As gene therapy vectors, transposons were found to have two advantages over viruses: first, clinical manufacture and quality control are easier, cheaper, and more reliable when viruses are employed. Second, unlike viral cargos, which are usually integrated into genes that can incur mutagenic risks, these SB transposons have few known preferences for integration sites [71]. The transposase can recognize the inverted repeat containing direct repeated sequences flanking the transgene (e.g., CAR) in a transposon [72]. The SB transposon is now employed in clinical practice and has exhibited promising antitumor efficiency [69]. To further enhance the transfection efficiency of the SB system, several new transposases such as SB10, SB11, and SB100X have been used in studies to deliver various genes into different cells [73].

In addition to vector systems, emerging genome editing technologies are also required for successful CAR-Ts immunotherapy. To date, four major platforms have been exploited for these site-specific DNA-editing purposes: meganucleases, zinc finger nucleases, transcription activator-like effector-nucleases, and most recently the clustered regularly interspaced short palindromic repeats (CRISPR/Cas) system [74].

## RATIONALITY OF CAR-T CELLS THERAPY IN OC

CAR-T cells combine both antigen specificity and T-lymphocyte activation properties in a single fusion molecule. During the past decade, CARs have demonstrated remarkable effects on patients with hematological tumors [75, 76]. Using them to target solid tumors, however, is challenging, probably because of features in their histopathological structure and their difficulty in T-cell trafficking and T-cell infiltration into tumor sites [77]. Solid tumors have special histopathological features, including poor integrity of issue structure, a high concentration of blood vessels, and extensive vascular leakage. These features induce selectively enhanced permeability and retention (EPR) of lipid particles and macromolecular substances in solid tumors. Effector T lymphocytes play critical roles in the success of T-cell-based immunotherapy. The EPR effect in a solid tumor microenvironment can impede the infiltration of effector T lymphocytes into tumor tissues [78]. Aberrant vasculature, the downregulation of adhesion molecules, and the mismatch of chemokine–chemokine receptor pairs may also lead to the poor homing of T lymphocytes [79]. Heterogeneity is a prominent feature of many types of solid tumor and causes specific CAR-Ts to become effective in only a portion of tumor cells [78]. Therefore, the most advantageous way to treat solid tumors using CAR-Ts is to identify the specific cell surface antigens. Furthermore, the immunosuppressive environment within a solid tumor also causes difficulties in the use of CAR-Ts. A hypoxic, low pH intrinsic microenvironment and the activated inhibitory pathways can induce or enhance immunosuppression within tumor sites [80]. Despite the several challenges to applying CAR-Ts to solid tumors, numerous CARs targeting diverse cancer types including OC have been developed [41, 81, 82]. Although clinical pilot trials have just begun in OC, the potential of this form of CAR-T-cell immunotherapy is becoming increasingly evident.

OCs are immunogenic tumors, and OC immunotherapies have demonstrated considerable potential for treating patients with such tumors [83]. The first and crucial evidence confirming the rationality of immunotherapy for OC was presented by a study revealing that CD3^+^ tumor-infiltrating T lymphocytes (TILs) were correlated with patients’ increased overall survival (OS) and progression-free survival [84]. The administration of autologous TILs to patients with OC after surgery and chemotherapy resulted in increased objective regression, prolonged disease-free survival (DFS), and improved survival rate, supporting that T-cell transfer therapy actively inhibits OC cell growth [7, 8]. A study on tumor microenvironments demonstrated that CD3^+^ and CD8^+^ T lymphocytes were vital antitumor effectors in OC [85]. By contrast, an increased number of CD4^+^CD25^+^FoxP3^+^ regulatory T cells in the OC microenvironment has been demonstrated to predict chemoresistance and poor prognosis [86, 87]. Furthermore, solid tumors often employ multiple mechanisms to attenuate the validity of T-lymphocyte-mediated attacks by in turn downregulating MHC class I (MHC-I) or other molecules related to the antigen-processing machinery in order to evade immune responses [88]. The downregulation of MHC-I on the surface of a cancer cell restrains the homing of T lymphocytes because the interaction between the TCR and peptide-MHC is a prerequisite for T-lymphocyte activation [89]. Nevertheless, CARs bypass the immune escape mechanism of cancer cells because they endow T lymphocytes with cytotoxic effector features in an MHC-unrestrictive manner [90]. This is particularly important for OC, in which the advanced stage is correlated with MHC downregulation [91]. These findings imply that patients with OC may benefit from CAR-T cells in clinical practice.

## ACTIVE CAR-T CELL THERAPY IN OC

Human T lymphocytes modified with synthetic receptors can redirect tumor antigens specifically and present striking efficacy in numerous human malignant tumors [92]. In addition, CAR-T cells targeting diverse tumor-associated antigens have already been developed, characterized, tested, and reported [93]. The cell surface antigens targeted by CARs include proteins, carbohydrates, and glycolipids [94]. The most common antigens targeted by CARs in OC include MUC16, folate receptor-α (FRα), mesothelin, and HER2 (Table 2).

**Table 2:** Active clinical trials of CAR-T immunotherapies for ovarian cancer*

Target antigen	Receptor type (other specificity)	Gene transfer vehicle	NCT	Phase	Sponsor	Indication
MUC16	4H11-28z/ fIL-12/ EFGRt	RVs	02498912	I	Memorial Sloan Kettering Cancer Center	Recurrent MUC16ecto^+^solid tumors
α-Folate receptor	n.p.	RVs	00019136	I	NCI	Advanced EOC
	ScFV- 4-1BB- CD3ζ	LVs	02159716	I	University of Pennsylvania	Patients with mesothelin expressing cancers
	n.p.	LVs	03054298	I	University of Pennsylvania	Patients with mesothelin expressing cancers
Mesothelin	ScFV- CD3ζ- CD137	RVs	02580747	I	Chinese PLA General Hospital	Relapsed and/or chemotherapy refractory malignancies
	n.p.	RVs	01583686	I, II	National Cancer Institute(NCI)	Metastatic cancer expressing the mesothelin molecule
HER2	ScFV- CD3ζ- CD137	n.p.	01935843	I, II	Chinese PLA General Hospital	Chemotherapy refractory HER2+ advanced solid tumors
	n.p.	n.p.	02713984	I, II	Zhi Yang	HER2^+^ cancer
EGFR	ScFV- CD3ζ- CD137	LVs	01869166	I, II	Chinese PLA General Hospital	Chemotherapy refractory EGFR^+^advanced solid tumors
CD133	ScFV- CD3ζ- CD137	RVs	02541370	I	Chinese PLA General Hospital	Relapsed and/or chemotherapy refractory advanced malignancies
CEA	n.p.	RVs	01212887	I	Cancer Research UK	Patients with advanced CEA positive tumors
NKG2D	n.p.	n.p.	03018405	I	Celyad	Various tumors expressing NKR-2
NY-ESO-1	n.p.	RVs	02366546	I	Mie University	Unresectable, refractory solid tumors expressing NY-ESO-1
MAGE-A4	n.p.	RVs	02096614	I	Mie University	Unresectable, refractory, metastatic or recurrent tumors expressing MAGE-A4
WT-1	n.p.	n.p.	00562640	I	Memorial Sloan Kettering Cancer Center	Recurrent or persistent advanced EOC

* ClinicalTrials.gov

EOC, epithelial ovarian cancer; LVs, lentiviral vectors; NCI, National Cancer Institute; n.p., not provided; RVs, retroviral vectors.

### MUC16 ecto

**Figure 2:**
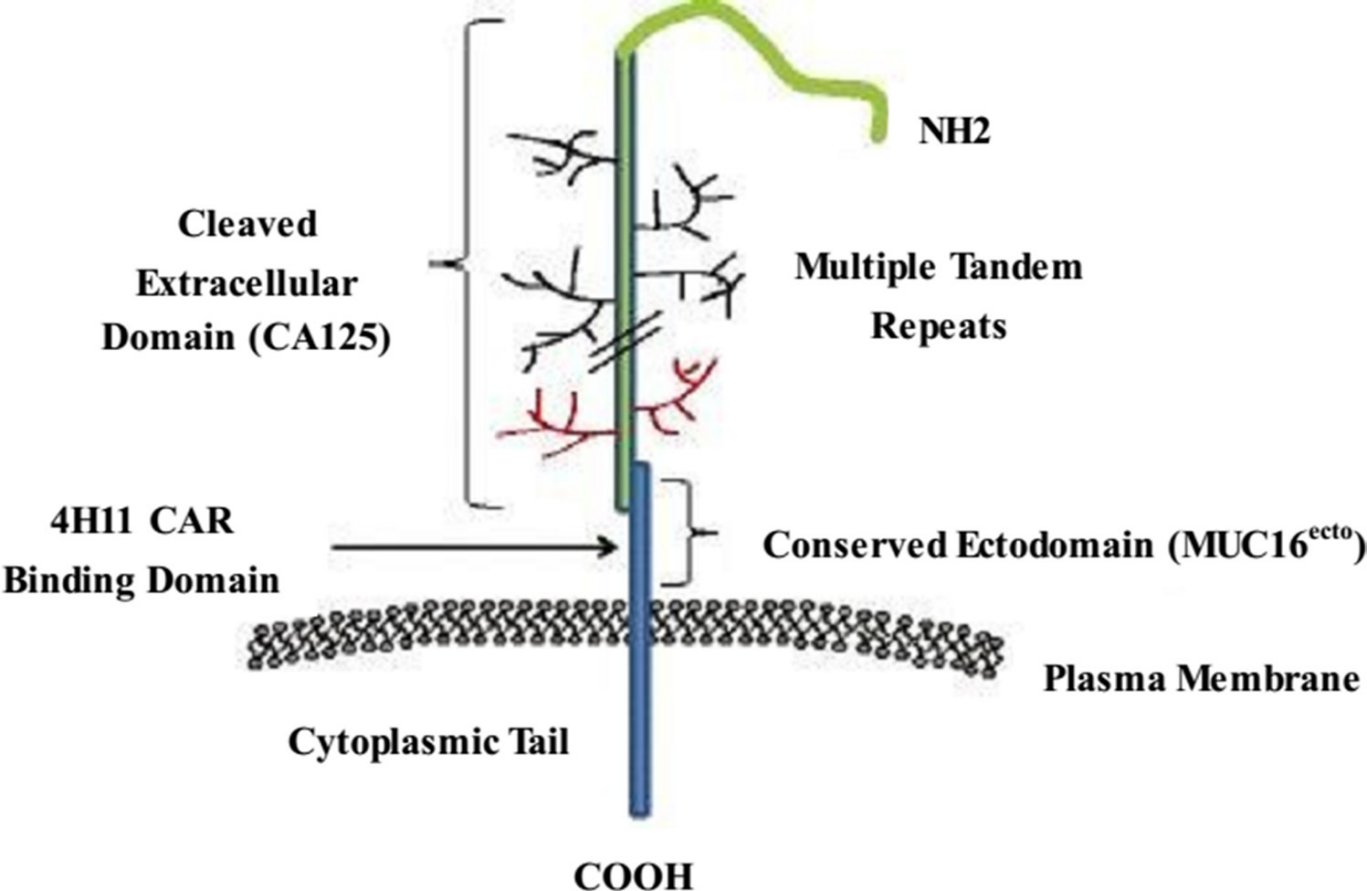
Schematic of MUC16 structure The full-length MUC16 contains a large cleaved and released domain named CA125 consisting of multiple repeat sequences, followed by a conserved cytoplasmic domain MUC16^ecto^ including a nonrepeating ectodomain, a transmembrane domain, and a cytoplasmic tail.

Mucins are vital biomolecules in cellular homeostasis and epithelial surface protection. MUC16 is a highly glycosylated mucin; it is overexpressed in most OCs and is an established surrogate blood biomarker (CA-125) for the diagnosis and progression of OCs [95, 96]. The full length of MUC16 comprises a large cleaved and released domain termed CA-125, which contains multiple repeat sequences; a conserved domain called MUC16 ecto, which contains a residual nonrepeating extracellular fragment; a transmembrane domain; and a cytoplasmic tail including a phosphorylation site (Figure 2) [97, 98]. A previous study suggested that MUC16 plays a role in OC cell metastasis, forming implants on the surface of the peritoneal cavity [99]. A hybridoma that generates an antibody specific to the extracellular conserved domain MUC16 ecto was used to produce a CAR specific to MUC16 ecto (4H11-28z); this CAR was then referenced to engineer autologous T lymphocytes targeted at a surface-exposed, retained antigen [10].

CAR-modified MUC16-target T lymphocytes have high MUC16-specific cytotoxic activity against OC cells *in vitro*. In addition, the infusion of expanded 4H11-28z-expressed T lymphocytes through intravenous infusion or intraperitoneal injection into mice bearing human MUC16+ ovarian tumors was discovered to either delay progression or completely eradicate tumors [100]. These investigational preclinical studies justify the in-depth investigation of MUC16-targeted T lymphocytes as a potential therapeutic strategy for patients with OC using high-risk MUC16+ tumor cells. Currently, a dose-escalation phase I trial using IL-12-secreting MUC16 ecto-directed CAR-T cells for recurrent OC is ongoing [10].

### Folate receptor-α (FRα)

FRα is a glycosylphosphatidylinositol-anchored protein that is overexpressed on the surface of epithelial tumors including breast, ovary, lung, colorectum, brain, and other solid malignant tumors, but the expression of which is limited in normal tissues [101]. A membrane-bound protein, FRα recognizes folic acid with high affinity and specifically mediates the cellular uptake of folic acid (and drug conjugates thereof) through receptor-mediated endocytosis [102]. Overexpression of FRα is related to high-grade cancer progression, poor prognosis in OC [103], and short survival rates in breast cancer [104]. Therefore, FRα is a potential candidate for targeted immunotherapy of epithelial-derived malignancies, particularly for epithelial OC (EOC), in which nearly 90% of tumor cells express FRα [105].

Human T cells modified to express CARs specific for FRα have been reported to exhibit effective antitumor activity *in vitro* and in animal models; however, their effects on clinic patients appear to be weak due to their inability to persist and home in on tumor sites [11, 106]. In 2006, the use of genetically redirected CAR-T cells for the treatment of FRα^+^ OC was first reported in a clinical trial [107]. In this trial, no tumor burden reduction was observed in any patient with OC. Polymerase chain reaction analysis revealed that modified T cells were present in the circulation in large numbers for the first 2 days after transfer; however, their number rapidly declined and the modified T cells were barely detectable after 1 month in most patients. The study concluded that a large number of gene-modified tumor-reactive T cells can be safely given to patients, but these T cells do not persist for a long period [107]. Another study constructed CAR-T cells containing the FRα-specific scFv MOv19 coupled to the CD3ζ chain signaling module alone (MOv19-ζ) or in combination with the costimulatory motif CD137 (4-1BB) in tandem (MOv19-BBζ). The study found that MOv19-ζ or costimulated MOv19-BBζ CAR-T cells secreted diverse proinflammatory cytokines such as interferon (IFN)-γ, IL-2, TNF-α, IL-4, and IL-10 and exerted their cytotoxic function when cocultured with FRα+ OC cells *in vitro*. However, only costimulated MOv19-BBζ CAR-T cells mediated tumor regression in immunodeficient mice with large, established FRα+ human OC [11]. Moreover, MOv19-BBζ CAR-T cells mediated tumor regression in models of metastatic intraperitoneal, subcutaneous, and lung-involved human OC [11]. The study overcame the weaknesses of previous CAR approaches by increasing the persistence of modified T cells *in vivo*, in addition to enhancing the accumulation of the cells at tumor sites and their antitumor potency.

### Mesothelin

Mesothelin is so named because of its expression in mesothelial cells [108]. This membrane glycoprotein is glycosylphosphatidylinositol-linked and is overexpressed on the surface of mesothelioma and OC cells as well as in malignancies of the lung, pancreas, and stomach [108–110]. Mesothelin originates from the precursor mesothelin protein, which is cleaved by a furin-like protease, and it is also referred to as C-ERC/mesothelin [111]. Compared with its low expression level in normal tissues, mesothelin is a promising target for the treatment of OC because it is expressed in 82% of serous epithelial OC cells [112]. The function of mesothelin is still not completely understood; however, mesothelin binds to CA125, suggesting that mesothelin may contribute to the peritoneal and pleural metastasis of OC [113]. In addition, one study demonstrated that mesothelin overexpression is related to chemoresistance, shorter DFS, and poor OS in patients with epithelial OC [114].

In an animal model, lentiviral CAR-T-mesothelin cells injected into mice intratumorally or intravenously led to a marked decrease in tumor size or eradication of tumors in mesothelin-expressing OC and mesothelioma [115]. In addition, a fully human antimesothelin scFv P4 was reported in a preclinical model. Primary human T cells expressing P4 CAR specifically produced proinflammatory cytokines (including IFN-γ, macrophage inflammatory protein-1α, TNF-α, and IL-2) and degranulated and exerted potent cytolytic functions when cultured with mesothelin-expressing tumors *in vitro* in a xenogenic model of human OC [42]. A phase I clinical trial of CAR-T directed against mesothelin was conducted (NCT02159716), and the preliminary results revealed that an infusion of CAR-mesothelin T cells was well tolerated without an off-tumor effect or cytokine release syndrome (CRS) in patients with mesothelioma, OC, and pancreatic cancer. Currently, numerous clinical trials evaluating diverse mesothelin-directed CARs (NCT03054298, NCT02580747, NCT01583686) are ongoing.

### HER2

The HER2 oncogene (also known as ERBB2 or neu) is located in the long arm of chromosome 17 and belongs to the epidermal growth factor receptor family [116]. The HER2 protein (185 kDa) is encoded by the HER2 proto-oncogene and is involved in the development and progression of OC [117]. This protein consists of a large extracellular domain, a hydrophobic transmembrane domain, and an intracellular domain with an ATP-binding tyrosine kinase domain and a carboxy-terminal domain [118]. HER2 has been demonstrated to be expressed in approximately 40% of EOCs, as evaluated using immunohistochemistry, and its overexpression has been reported to be associated with poor prognosis in breast cancer, OC, and many other malignancies [117, 119–121]. Since the 1980s, academic laboratories have been increasingly producing monoclonal antibodies to target HER2 [119]. The development and application of Trastuzumab (Herceptin) mAb treatment represented a paradigm shift from nonspecific chemotherapy to molecularly targeted therapy in oncology. However, Trastuzumab is expensive and can cause side effects including cardiotoxicity, corneal ulceration, and neutropenia; hence, its use is limited [122–124]. Currently, HER2-specific CAR-T cells have been applied in preclinical studies. A study developed a novel, humanized HER2 CAR that contains a chA21 scFv region of antigen-specific mAb and T-cell intracellular signaling chains composed of CD28 and CD3ζ in tumor-bearing mice. Results revealed that the novel chA21 scFv-based, HER2-specific CAR-T cells recognized and killed HER2+ breast and OC cells *ex vivo* [125]. In a glioblastoma model, tandem CAR-T cells targeting HER2 and IL13Rα2 were discovered to mitigate tumor antigen escape, exhibit enhanced antitumor efficacy, and improve animal survival [126]. To date, clinical trials of HER2-specific CAR-T-cell therapy in OC have been rare. Ongoing clinical trials are listed in Table 2.

## CAR-RELATED TOXICITIES

Despite its potential and promising clinical results, CAR-T-cell immunotherapy involves several toxicities because of the presentation of tumor-associated antigens by healthy tissues and the inability to control T-cell activity. The prominent toxicities of CAR-T-cell immunotherapy are CRS and on-target, off-tumor toxicities.

The CRS effect (so-called cytokine-associated toxicity) is caused by intense tumor-killing actions mediated by numerous activated lymphocytes (B cells, T cells, and NK cells) [127]. The extremely high levels of cytokines such as C-reactive protein, IL-6, and IFN-γ observed in human bodies with cancer are several hundred times higher than the baseline levels, and such high levels typically cause clinical syndromes including hypotension, fever, and neurological changes and can even lead to sudden death. In clinical practice, gynecological oncologists reported CRS in a 52-year-old woman with OC who had received treatment using autologous mesothelin-redirected CAR-T cells (CART-meso). High-volume production of pleural fluid was evident after T-cell infusion, with a higher number of CART-meso cells in the pleural cavity compared with that in the blood, as well as a greater increase in IL-6 within the pleural fluid [128]. To enable assessment of the severity of CRS, Lee et al. published a grading system containing five grades that is based on the clinical signs and symptoms of CRS [129].

One previous study demonstrated that tumor-associated antigens were expressed not only in tumor cells but also in normal cells [130]. When tumor-associated antigens are used as targeting molecules for CAR-T-cell therapies, normal cells can also be distinguished and attacked by lymphocytes, causing damage to normal tissue (referred to as on-target, off-tumor toxicity). This type of on-target toxicity leads to rapid cardiopulmonary toxicity and can even be life-threatening. A case report detailed a serious “on-target” adverse event following the application of T cells transduced using a CAR recognizing ERBB2. A 39-year-old woman with colon cancer metastatic to the lungs and liver experienced respiratory distress, and a drastic pulmonary infiltrate was identified on a chest X-ray after CAR-T-cell therapy. She was intubated, and despite intensive medical intervention, she died 5 days after treatment. The death of this patient was speculated to be a result of the infusion of highly active anti-ERBB2-directed CAR-T cells, which recognized ERBB2 expressed by normal lung epithelial cells and released inflammatory cytokines (TNF-α and IFN-γ) that caused pulmonary toxicity and multiorgan failure [131].

Although CAR-T therapy involves toxicities when used to treat solid tumors including OC, these toxicities are generally much weaker than those when blood cancers are the target. Because immunity in a solid tumor is more restricted to a local site, the T-cell trafficking and infiltration into tumor sites is more difficult, and T-cell functions are inhibited in cancer microenvironments by proteins or immunosuppressive cells [77]. In addition, great attention must be paid to CAR-T related toxicities in solid tumors because of the risk of mortality they incur.

## SOLUTIONS FOR THE MITIGATION OF CAR-RELATED TOXICITIES

To mitigate CAR-related toxicities, several methods have been developed either in OC or other malignancies. Herein, we discuss common solutions for CAR-related toxicities applied to solid tumors including OC and detail innovative approaches that can indicate some future research directions for its treatment of OC. In clinical practice, to moderate the immunotoxicities caused by therapeutic CAR-Ts, exogenous inhibitors with cytostatic or cytotoxic effects such as corticosteroids and cytokine blockades (tocilizumab and etanercept) were successfully used to ameliorate CRS [132, 133]. To reduce on-target, off-tumor toxicity, a safe, effective, and widely used method of improving safety and efficacy is to incorporate a regulated suicide gene into engineered CAR-T cells, such as the HSV-TK [134] and iCasp9 [135] suicide genes. An ideal suicide gene, which should eliminate CAR-T cells, should be stably coexpressed in the modified cells in addition to being in sufficiently high levels to elicit cell death. Therefore, a suicide gene should exhibit high specific activity and low susceptibility to endogenous antiapoptotic molecules. Ad5.SSTR/TK.RGD is an infectivity-enhanced adenovirus expressing a therapeutic thymidine kinase suicide gene and a somatostatin receptor. One study demonstrated the feasibility, safety, and potential clinical efficacy of suicide gene therapy by using Ad5.SSTR/TK.RGD in OC [136]. CTLA-4, PD-1 immune inhibitory receptors, and inhibitory-CAR-engineered T cells edited using a system with CRISPR may also have similar effects [26]. Furthermore, to overcome potential on-target, off-tumor adverse events, identifying genuine tumor-specific targets is imperative. An emerging method of improving the safety of CAR-T cells is the use of a dual-antigen receptor, which involves the coexpression of two different antigen receptors to target two different tumor antigens [137]. CAR-T cells with dual-antigen receptors have been reported to have high specificity and accuracy and to induce less intense side effects. Moreover, identification of appropriate and specific tumor-associated antigens expressed only in cancer cell membranes, but not in normal cells, is imperative. In addition, oxygen-sensitive CAR-T cells, which can restrict the immune response to tumor tissue, are safer because they minimize on-target, off-tumor effects. As mentioned, tumor microenvironments have been associated with hypoxia. Juillerat et al. [138] generated CAR-Ts that were responsive to an hypoxic environment by fusing an oxygen-sensitive subdomain of HIF1α to a CAR scaffold. They discovered that increased surface expression of HIF-CARs along with improved cytolytic properties of T cells can be obtained under hypoxic conditions.

## DISCUSSIONS AND CONCLUSIONS

CAR-T cells have two major advantages compared with TCR-T cells: (1) MHC-independent recognition of tumor-associated antigens, which enables the extensive application of CARs irrespective of the patient's MHC and the successful recognition of cancer cells with downregulated MHC expression, and (2) extremely low risk of mispairing with TCRs endogenously. CAR-T cells are therefore not just an alternative but may prove to be superior to TCRs as a therapeutic strategy. The use of CAR-T cells in cancer immunotherapy is currently coming of age. As previously mentioned in our description of the clinical application of CAR-T-cell immunotherapy in OCs, CAR-T-cell immunotherapy is an appealing area of OC research, expands the spectrum of antitumor strategies, and has both clinical and economic value. Increasing numbers of clinical trials are being performed to verify its safety and efficacy in OCs (NCT02159716, NCT03054298, NCT02580747, and NCT01583686). Moreover, various combinations of CARs with TCRs are promising, achieving greater persistence and response. However, the potential side effects and cytotoxicities including the CRS effect and “on-target” adverse events must still be considered and resolved before this novel and promising approach can be broadly applied to treat patients with OC. Furthermore, a thorough understanding of the molecular mechanisms during the design, processing, and implementation steps is crucial to improve the safety and efficacy of CAR-T cell immunotherapy.
